# Professional Handwriting

**Published:** 1916-02-12

**Authors:** 


### PROFESSIONAL HANDWRITING.

The medical profession has many critics, for its work has frequent public relations, and not all its policies and standards are understanded of the people. On the whole, the profession is probably treated very fairly, and if sometimes it receives blame which it has not merited there are other occasions on which the flag-waving is in excess of the virtues. Hence in the end the balance is probably not far removed from justice, while for the individual practitioner we should say that fate leans towards generosity. He, on his part, if not ready with a frolic welcome for every comment, manages on the whole to take his critics with a light heart. If he is wise, too, he learns from the enemy.

Indistinct handwriting appears rather a juvenile ground for censure, but there are existing circumstances which give to the matter a special degree of prominence. The first of these is certain legislative enactments which have multiplied the occasions on which professional documents come before public authorities; only a few days ago one of the Metropolitan magistrates openly regretted what he called the habit of doctors to write in an illegible fashion. A second influence which has increased impatience towards indistinct handwriting is the widespread use of the typewriter. A few years ago it was the common lot to struggle with hieroglyphics; they were as frequent as the postman, and to attempt their interpretation was a daily discipline. But nowadays they are the exception, and, standing in sharp contrast with the clear work of the typewriter, the full measure of their abominations is made manifest. What was formerly regarded as in the very nature of things is now seen to be a wholly gratuitous outrage. In these circumstances the limits of human endurance have shrunk to a smaller circle and the explosion point is correspondingly close at hand. That doctors are the only criminals no one will say; sometimes, indeed, they are the victims. But the business letter usually has the name of the writer printed at the head of it, and hence any strange contortions at its close provoke no wrath. The example might well be followed by those of our *confrères* whose habits are beyond repair.

In addition to the public relations of certain professional documents, it has to be remembered that prescriptions and similar writings concern matters of health and even of life, and there is thus a special claim for distinctness. Pharmacists often cultivate great dexterity in deciphering such writings, but they ought not to be put to this responsibility, and in any event the need for it does not increase respect for the profession. An old writer on prescriptions speaks of certain faults in them as likely to provoke "the sneering of the apothecary's boy," and, though worse penalties are possible, even this one is just as well avoided. In some quarters it seems to be regarded as an undignified thing to write distinctly, but the real explanation of the defect is much more elementary. The fixed and perhaps irremediable habit of to-day is the natural outgrowth of earlier laziness and carelessness. This is to put the matter in plain language which, if it does not mend the seniors may at least warn the juniors. If nought else will avail, our contortionists ought to take to the typewriter. Their periodic puzzles are beyond the patience of the twentieth century.

